# Quis Custodiet Ipsos Custodes (Who Controls the Controllers)? Two Decades of Studies on HDAC9

**DOI:** 10.3390/life11020090

**Published:** 2021-01-27

**Authors:** Claudio Brancolini, Eros Di Giorgio, Luigi Formisano, Teresa Gagliano

**Affiliations:** 1 Department of Medicine, Università degli Studi di Udine, p.le Kolbe 4, 33100 Udine, Italy; eros.digiorgio@uniud.it (E.D.G.); teresa.gagliano@uniud.it (T.G.); 2 Department of Neuroscience, School of Medicine, “Federico II” University of Naples, Via Pansini, 5, 80131 Naples, Italy; luigi.formisano@unina.it

**Keywords:** class IIa HDACs, MEF2, MITR, cardiovascular disease, inflammation, HDAC9, MEF2D

## Abstract

Understanding how an epigenetic regulator drives different cellular responses can be a tricky task. Very often, their activities are modulated by large multiprotein complexes, the composition of which is context- and time-dependent. As a consequence, experiments aimed to unveil the functions of an epigenetic regulator can provide different outcomes and conclusions, depending on the circumstances. HDAC9 (histone deacetylase), an epigenetic regulator that influences different differentiating and adaptive responses, makes no exception. Since its discovery, different phenotypes and/or dysfunctions have been observed after the artificial manipulation of its expression. The cells and the microenvironment use multiple strategies to control and monitor HDAC9 activities. To date, some of the genes under HDAC9 control have been identified. However, the exact mechanisms through which HDAC9 can achieve all the different tasks so far described, remain mysterious. Whether it can assemble into different multiprotein complexes and how the cells modulate these complexes is not clearly defined. In summary, despite several cellular responses are known to be affected by HDAC9, many aspects of its network of interactions still remain to be defined.

## 1. Class IIa Histone Deacetylases (HDACs): A Short Introduction

The regulation of the access to DNA is a key aspect in the control of gene expression. In general terms, epigenetics investigates the mechanisms through which this access is modulated by the cells and the environment. There are several controllers, which exhibit different biochemical activities (principally on DNA and histones) in order to sculpture the epigenome. The final goal is to allow or deny access to DNA. Histone deacetylases (HDACs) are important epigenetic regulators (controllers) that when acting on histones aim to reverse an open state of the chromatin. HDAC9 belongs to the class IIa HDACs subfamily of deacetylases that in vertebrates show a poor/absent enzymatic activity towards acetyl-Lys due to the tyrosine/histidine substitution in the catalytic pocket [1]. Nonetheless, they are equally powerful repressors of transcription. Class IIa HDACs are recruited by specific transcription factors (TF) on the regulative elements of different genes to silence their expression [2]. On chromatin, in virtue of their ability to assemble into multiprotein complexes, class IIa HDACs engage other transcriptional co-repressors and can reset the epigenetic signature [3,4]. In addition, class IIa HDACs have also been reported to modulate the acetylation of non-histone proteins [5]. Environmental signals control class IIa HDACs’ activities through different strategies, including regulation of transcription and translation, ubiquitin-dependent degradation, and selective proteolysis [2,6,7,8]. A quick option for modulating class IIa HDACs repressive potential is operated through the control of their subcellular localization. These deacetylases shuttle in and out from the nucleus in a phosphorylation-dependent manner. Class IIa HDACs have a set of conserved serines that, once phosphorylated, become docking sites for 14-3-3 chaperone conserved serines that, once phosphorylated, become docking sites for 14-3-3 chaperone proteins, which in turn escort the deacetylases from the nucleus into the cytoplasm. This control limits their repressive influence [2,3,4,9,10]. On the other hand, phosphatases such as PP2A can promote HDAC nuclear import and consequently gene repression [11].

## 2. HDAC9: An Historical Perspective

HDAC9 was originally identified as a MEF2 (myocyte enhancer factors 2) partner via a yeast two-hybrid screen [12]. Indeed, a shorter isoform termed MITR (MEF2-interacting transcription repressor), lacking the carboxy-terminal HDAC domain was initially identified. MITR is able to repress MEF2 transcription and its expression is related to muscle development. Starting at the neurula stage within the mature somites, MITR expression is subsequently restricted to the myotomal muscle. Despite missing the deacetylase domain, MITR represses MEF2 activity, since it can assemble into an actively deacetylating complexes. The recruitment of HDAC1 to MEF2 was proposed as the molecular mechanism mediating the repressive influence of MITR [12].

**Figure 1:**
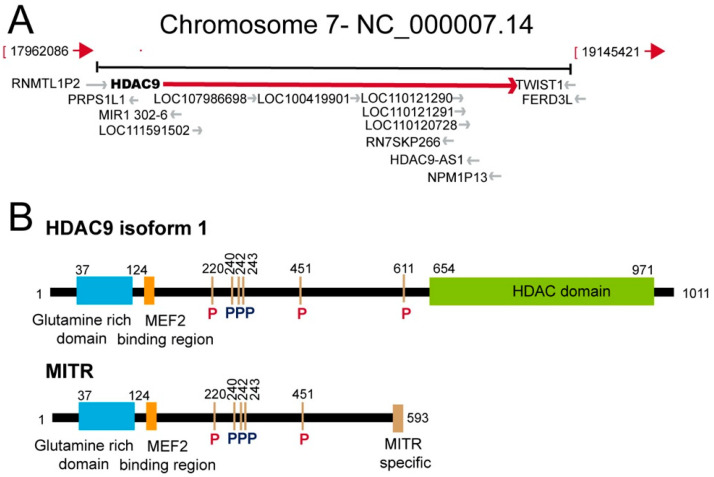
The HDAC9 (histone deacetylase) genomic locus and schematic representation of the HDAC9 protein and of the splicing variant myocyte enhancer factors 2-interacting transcription repressor (MITR). (**A**) Simplified view of the HDAC9 genomic region including adjacent genes. Data are from assembly GRCh38.p13 (GCF_000001405.39). (**B**) Schematic representation of HDAC9 and of its splicing variant MITR. The main domains/regions are indicated. Phosphorylation of 14-3-3 binding sites are highlighted in red. Other phosphorylation sites are in black [2]. The HDAC9 isoform 1 from *Homo sapiens* was selected for the analysis (accession: AAK66821.1).

In parallel, HDAC9 was identified by a second research team as HDRP (HDAC-related protein). HDRP shares 50% identity in the amino acid sequence to the non-catalytic N-terminal domain of HDAC4 and 5 and acts as a repressor of transcription, by recruiting both HDAC1 and HDAC3 [13]. The recruitment of the COOH-terminal-binding protein (CtBP), through a CtBP-binding motif (P-X-D-L-R) conserved in MITR and in the other class IIa HDACs is another mechanism used to exert the repressive pressure [14]. MITR lacks also the nuclear export signal that lies in the C-terminus of class IIa HDACs (Figure 1). As a consequence, it frequently accumulates in the nucleus. However, during muscle differentiation, its repressive action is switched-off by the CaMK-dependent phosphorylation of the 14-3-3 binding sites (Se-218 and 448) [15]. Although the heterodimerization of MITR with other class IIa paralogues could theoretically lead to its cytoplasmic export, the binding of 14-3-3 is sufficient to turn off the repressive activity of HDACs regardless of any cytoplasmic relocation [16,17]. It is evident that, the comprehension of the fine regulation of HDAC9/MITR nuclear/cytoplasmic shuttling as well as of the switching-on/off dynamics, deserve further studies, possibly by applying CRISPR (Clustered Regularly Interspaced Short Palindromic Repeats)-based strategies to monitor the shuttling of the endogenous proteins. 

**Figure 2:**
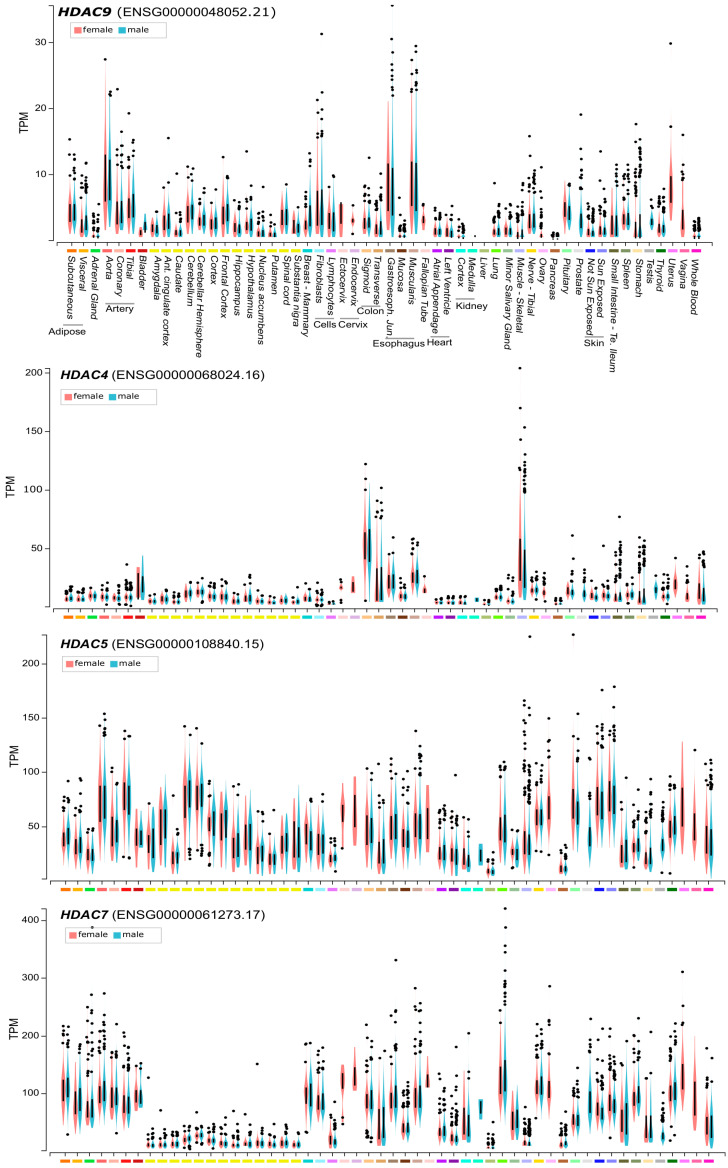
HDAC9 and class IIa HDACs mRNA expression levels in different human tissues. Lymphocytes are EBV (Epstein-Barr virus)-transformed and fibroblasts were grown in culture. Basal ganglia were analyzed in the case of caudate, nucleus accumbens, putamen. The yellow bars indicate different regions of the central nervous system. Expression values are shown in TPM (transcripts per million) calculated from a gene model with isoforms were collapsed to a single gene. Box plot are shown as median and 25th and 75th percentiles. Points are displayed as outliers if they are below or above 1.5 times the interquartile range. Data are from https://www.gtexportal.org.

Two years later the discovery of MITR, the full-length HDAC9 containing the deacetylase domain was identified. The genomic characterization of the *HDAC9* locus on human chromosome 7p21, revealed the existence of different splicing isoforms (including MITR) that show tissue specific expression [18,19]. The *HDAC9* gene extends up to 458 kb. HDAC9 protein is constituted by 1069 aa and the relative mRNA by 26 exons. *HDAC9* can be subjected to alternative splicing, thus yielding to several variant isoforms not limited to *MITR*. At least, 29 isoforms have been identified so far. *HDAC9* and *HDAC9a* isoforms have 24 and 20 exons, respectively. The deacetylase domain is located between the 12th and 22nd exons and is highly conserved among the different class IIa HDACs. The *HDAC9* promoter lacks TATA, CCAAT boxes and CpG-rich elements [18,19,20]. These features explain the low basal expression of *HDAC9* observed in different tissues (Figure 2). HDAC9 could also be part of protein complexes aimed to coordinate the deposition of different repressive marks, as indicated by the interaction with the heterochromatin protein 1 [21].

Mice mutant for *Hdac9* are sensitized to hypertrophic signals and exhibit stress-dependent cardiomegaly. This result confirmed the role of the MEF2-class IIa HDACs axis in the regulation of the transcriptional program governing cardiac hypertrophy and heart failure [22]. As explained above class IIa HDACs activities are modulated by phosphorylation-dependent nuclear-cytoplasmic shuttling (Figure 1B). Hypertrophic signals use a non-classic CaMK HDAC9-kinase to unleash MEF2 transcription [22]. In cardiomyocytes, cAMP, through (PKA)-dependent inhibition of PKD promotes the nuclear accumulation and blunt phosphorylation at the 14–3-3 binding sites (Ser218/448) of HDAC9 [23]. Protein kinase C-related kinases (PRKs) can phosphorylate the serine 253 in the NLS of HDAC9/MITR. This site is conserved in HDAC5 and HDAC7 but is absent from HDAC4. It was suggested that its phosphorylation could impair the nuclear import of the deacetylases, by acting as priming step for further phosphorylations [2,24,25].

Comparative ChIP-seq (chromatin immunoprecipitation and sequencing) studies have unveiled that HDAC9 can bind genomic regions also independently from MEF2. Despite these experiments provide a static view of the interactions of a protein with the DNA, they suggest that class IIa HDACs can assemble into different complexes that interact with different genomic regions [6,26]. When compared to the other family members, HDAC9 is the lowest expressed in normal human adult tissues [27]. Higher mRNA levels are present in blood vessel, esophagus and uterus, all tissues characterized by the abundance of smooth muscle cells (Figure 2). This very low level of expression suggests that HDAC9 transcription could be under the tight control of environmental signals. Therefore, even though the regulation of nuclear/cytoplasmic shuttling seems to be the most commonly used strategy to govern class IIa HDACs activities, in the case HDAC9 the transcriptional control could also play a prominent role.

## 3. Regulation of HDAC9 Expression during Differentiation: The Adipogenesis and the Risk of Diabetes

As discussed above, in addition to post-translational regulations, it is highly plausible that the mRNA levels of *HDAC9/MITR* are subjected to intense regulations in different contexts. In mesenchymal stem cells (MSCs), MITR is the master switcher between the osteogenic and the adipogenic differentiation. The Polycomb repressive complex 2 (PRC2) subunit EZH2, which catalyzes the tri-methylation of H3K27, binds the *HDAC9/MITR* promoter and represses its transcription. This repressive pressure is operative in adipocytes but not in osteoblasts. Silencing of MITR inhibits osteogenesis and enhances adipogenesis. The suggested mechanism relies on the interference of HDAC9 with PPARγ-2 activities and particularly on its capacity of binding target genes’ promoters [28]. HDAC9 can also inhibit osteoclast differentiation and its expression is down-regulated during osteoclastogenesis. In agreement with this result, *Hdac9^-/-^* mice are also characterized by elevated bone resorption and lower bone mass [29].

A role of HDAC9 in adipogenesis was confirmed by further studies. Among the different HDACs, HDAC9 is specifically down-regulated before adipogenic differentiation and preadipocytes from *Hdac9^-/-^* mice exhibit accelerated adipogenesis. The research team discovered that in preadipocytes HDAC9 is recruited with USF1 at the E-box region of the *C/EBPα* gene promoter, a master regulator of adipocyte differentiation. Here HDAC9 represses *C/EBPα* transcription [30]. Indeed, the link between HDAC9 and USF1 was proposed by an earlier study. During fasting HDAC9 accumulates in the nuclear compartment and USF1 is deacetylated. The molecular basis of this deacetylation is unknown, but it is pivotal to down-modulate the transcription of the fatty acid synthase (*FAS*), a central enzyme in lipogenesis [31]. *HDAC9* mRNA levels can be regulated also at post-transcriptional levels. In bone marrow mesenchymal stem cells, *miR-188* is an important regulator of the switch, between osteogenesis and adipogenesis, occurring with aging. *miR-188* directly regulates HDAC9 and RICTOR (RPTOR independent companion of MTOR complex 2) [32]. Overall these data confirm that HDAC9 is a key factor in this differentiating switch.

The activity of HDAC9 during the adipogenic differentiation could have clinical relevance. In mice subjected to chronic high-fat diet (HFD) adipogenic differentiation is impaired and Hdac9 is expressed at high levels. Removal of *Hdac9* improves adipogenic differentiation, glucose tolerance and insulin sensitivity, and reduced hepatosteatosis. *Hdac9^-/-^* mice subjected to an HFD are characterized by upregulated expression of the beige adipocyte marker genes, increased energy expenditure and adaptive thermogenesis [33]. 

Cholesterol-lowering statins increase type 2 diabetes (T2D) risk, possibly by affecting adipogenesis and insulin resistance. Within this vicious cycle a dysregulated expression of HDAC9 emerges. DNA methylation represents a key epigenetic modification to repress gene expression through the organization of a compact chromatin status. Hypomethylation of the HDAC9 promoter region is elicited by statins (atorvastatin and mevastatin) though an undefined mechanism. This hypomethylation correlates with an increased *HDAC9* mRNA expression. Higher levels of HDAC9 cause the transcriptional repression of *ABCG1*, a cholesterol efflux gene. Artificial silencing of ABCG1 reduces the expression of key genes involved in adipocyte differentiation and decreases insulin signaling and glucose uptake [34].

Obesity is a consistent risk for developing metabolic diseases including diabetes. Additional indications point to a possible contribution of HDAC9 to diabetes. HDAC9 is expressed in insulin-producing β-cells and Hdac9 loss-of-function enhances β-cell mass when analysed at E18.5 and P7. This increase is not due to hypertrophy but rather to a burst in differentiation [35]. HDAC9 expression can be strongly induced upon hepatitis C virus (HCV) infection. Here, HDAC9, through the upregulation of gluconeogenic genes, seems to be involved in the development of HCV-associated exaggerated gluconeogenic responses. This role could suggest a contribution of HDAC9 to the development of type 2 diabetes [36]. Since the silencing of HDAC9 increases the acetylation of FOXO1 TF, a well-known regulator of gluconeogenic TFs, the FOXO1-HDAC9 axis could explain the positive effect of HDAC9 on gluconeogenesis [37]. Another link between HDAC9 and diabetes, metabolic and cardiovascular risks, has been proposed through the control of the circulating levels of the adipocyte-secreted adiponectin. Higher serum levels of adiponectin are associated with protection against inflammation and with a lower risk for obesity, cardiovascular disease, and type 2 diabetes. A genome-wide association study has identified an association between high adiponectin levels and a risk allele that may reduce HDAC9 expression or function [38].

In muscles the neuronal activity represses HDAC9 expression. The existence of this pathway was proved by the up-regulation of HDAC9 levels after denervation. Unfortunately, how neurons impact on muscle HDAC9 has not been characterized [39,40]. In the central nervous system (CNS) HDAC4 and HDAC5 have been intensively investigated for the ability to regulate different adaptive responses such as addiction, plasticity and memory [41,42,43,44]. It is possible that HDAC9 could be a further regulator of CNS functions. In fact, the *miR-183/96/182* cluster can target *Hdac9* and several plasticity-related genes. When this cluster is up-regulated in the hippocampus of adult mice it enhances object memory, whereas its down-regulation impairs this memory [45].

## 4. HDAC9 in Atherosclerotic Plaques and Cardiovascular Disease (CVD)

Atherosclerosis is a complex disease under the influence of both genetic and environmental factors. Atherosclerotic plaque formation, destabilization and an eventual rupture is the source of several cardiovascular diseases, including myocardial infarction and stroke [46]. The initial evidences of an association between *HDAC9* and CVD were reported in 2012. The *HDAC9* SNP (single nucleotide polymorphism) (rs11984041) was positively associated with large artery stroke (LAS) in the European population [47]. The mechanisms though which HDAC9 can increase stroke risk were not defined, as well as its consequence on HDAC9 activities. This SNP lies within the last large intron at the 3’ end of the gene (Figure 1A). The authors hypothesized a role of the SNP in accelerating atherosclerosis. Soon afterwards, a second SNP in bone marrow mesenchymal stem cells (rs2107595) was associated with LAS [48]. This SNP lies close to *HDAC9* loci in the intergenic region between *HDAC9* and *TWIST1* loci (Figure 1A) A subsequent study associated these SNPs with common carotid intima-media thickness and with the presence of carotid plaque [49]. An analysis of *HDAC9* SNPs in the Chinese population identified two additional intronic variants (rs2389995 and rs2240419), associated with LAS risk. The authors discussed about a possible heterogenic effect of the *HDAC9* SNPs in different ethnic populations [50]. A further SNP, in the 3′UTR of *HDAC9* (rs2023938), was also associated to LAS [51]. In summary, these SNPs are the smoking gun for a role of HDAC9 in the homeostasis of the cardiovascular system. Expression studies, in isolated peripheral blood mononuclear cells, for the intergenic rs2107595 allele were performed. These analyses highlighted an increase of *HDAC9* mRNA levels, but not of those of the neighboring genes (*TWIST1/FERD3L*) [52]. A similar observation was reported by Wang and co-authors [53]. A more recent study, performed on whole blood cells, did not evidence differences in *HDAC9* levels in the presence of the risk allele. Nevertheless, changes in the expression of genes controlling lipid metabolism and inflammation, between the two groups, were observed [54]. Importantly, several people that carry the risk allele rs2107595 do not have stroke, thus indicating the contribution of additional factors.

In agreement with the involvement of HDAC9 in LAS, *Hdac9^-/-^ApoE^-/-^* mice compared to *Hdac9^+/+^ApoE^-/-^* mice exhibit markedly reduced lesion sizes throughout atherosclerotic aortas and significantly less advanced lesions. However, the analysis of human atherosclerotic plaques revealed no association between this SNP and specific plaque characteristics [52]. Overall, these discoveries have fueled the research on HDAC9 in CVD. Soon after that it was reported that HDAC9 is up-regulated in the ischaemic cerebral hemisphere after cerebral ischaemia/reperfusion (I/R) injury. In this rat model, Hdac9 silencing attenuates cerebral injury in ischaemic stroke. During oxygen-glucose deprivation, the inflammatory response and apoptosis of brain microvessel endothelial cells were reduced, in the presence of low levels of Hdac9 [55]. Interestingly, the class IIa HDACs inhibitor TMP269, by counteracting stroke-induced HDAC9 increase, reduces neuronal cell death and ameliorates general and neurological scores [56].

HDAC9 was further recognized as an important risk factor for myocardial infarction, coronary artery disease, peripheral artery disease and atherosclerotic aortic calcification [57,58,59,60]. In atherosclerotic aortic calcification, increased expression of HDAC9, in human aortic smooth muscle cells, promotes calcification and reduces contractility, while its inhibition hinders calcification and enhances cell contractility. The authors proposed that the action of HDAC9 on vascular calcification could be the unifying molecular pathogenetic mechanism that is responsible also for the other CVD. They reported a reduction of Runx2 expression in the absence of Hdac9 [60].

Additional work has been performed to investigate the molecular mechanisms behind the most important risk variant of *HDAC9*: the rs2107595. The genomic region comprising this variant is characterized by a regulative “open” chromatin status, being enriched for DNase I hypersensitive sites, H3K27ac, H3K4me1, and H3K9me3. Undoubtedly, the presence of such epigenetic markers points to a regulative function. More specifically, the SNP alters a consensus binding site for the E2F3 TF. Indeed, E2F3 and pRb have an antagonistic effect on HDAC9 expression in HeLa cells. Importantly, macrophages homozygous for the risk allele show increased HDAC9 expression compared with macrophage homozygous for the common allele. A short fragment (41 bp) including the SNPs can drive transcription and the risk allele shows a stronger effect. Genome editing in Jurkat cells confirmed the higher potency of the A/A risk allele compared to the G/G common allele. This effect on transcription is cell and context dependent [61]. Since the risk allele abolishes the binding of E2F3, we can hypothesize that the repressive influence of pRB through chromatin looping reverberates at the promoter level. A control of pRB on HDAC9 transcription could also explain the oncogene-induced expression of the deacetylase (see below).

HDAC9 action on CVD can be mediated by genes under its regulation and that control the inflammatory response and/or the cholesterol efflux [62]. Recently, in large vessel atherosclerotic stroke patients an association between the *HDAC9* rs2107595 risk allele and the overexpression of genes involved in IL-6 signaling, leukocyte recruitment, chronic inflammation, cholesterol efflux, and platelet aggregation, has been found [54,63]. Accordingly, another paper demonstrated that the presence of *HDAC9* rs2107595 and *HDAC3* rs11741808 polymorphisms in combination with diabetes mellitus worsens atherosclerosis and causes stroke [64].

The contribution of HDAC9 to atherosclerosis could also stem from a modulation of inflammatory cells. In lethally irradiated *ApoE^-/-^* mice reconstituted with bone marrow from *Hdac9^+/+^ApoE^-/-^* or *Hdac9^-/-^ApoE^-/-^* mice, the absence of Hdac9 in bone marrow cells is atheroprotective. *Hdac9^-/-^* macrophages are characterized by a reduction in the Tnf-α-dependent up-regulation of pro-inflammatory cytokines and chemokines. Hdac9-deficient macrophages show reduced Tnf-α-induced phosphorylation of the Nf-kb subunit p65, which results in a reduced nuclear accumulation and transcriptional activity. The authors propose that HDAC9 forms a complex with Ikkα and Ikkβ resulting in their deacetylation and activation. Through this mechanism HDAC9 could control Nf-kb activities [65]. It is important to underline that these results were observed after the overexpression of the tagged proteins in HEK293 cells.

Hereditary thoracic aortic aneurysm (TAA) can be mediated by genetic alterations in elements of the TGF-β (Transforming growth factor-beta) pathway or in components of the vascular smooth muscle (VSM) contractile apparatus [66]. HDAC9 could represent the link between these two groups of inherited diseases. HDAC9 is up-regulated in experimental conditions recapitulating TAA. The initial investigations focused on the mRNA levels and the researchers have not clarified which *Hdac9* isoform is up-regulated in their TAA model. Certainly, impacting on MITR levels and the use of *Hdac9*-deficient animals strongly suggest that some HDAC9 isoform could influence the abnormal phenotypes of VSM cells in models of TAA [67]. Additional studies suggest that HDAC9 could also be involved in coronary and cerebrovascular stenotic disease. *Hdac9* expression is significantly up-regulated in mice after carotid arterial ligation. A condition that triggers neointimal changes such as remodeling of the extracellular matrix, VSM cells proliferation and inflammation. 21 days after ligation *Hdac9*-deficient mice evidenced significantly reduced neointimal hyperplasia and stenosis [68].

## 5. HDAC9 Expression in Inflammation and in the Immune Response

The aforementioned variances in Nf-kb activation, observed in the absence of Hdac9, point to a role of this epigenetic regulator in inflammation and the immune responses. The generation of *MRL/lpr* mice with *Hdac9* deficiency and the appearance of aberrant effector T cell function, confirmed the role of Hdac9 in lympho-proliferation, inflammation and autoantibody production. The deficit of Hdac9 conferred a survival advantage and an increased systemic production of Il-4. In the absence of Hdac9, in vitro stimulated CD4+ T cells (using anti-CD3 and anti-CD28 mAbs), secrete significantly less Il-2 and Il-12 and show mild decreases in Ifn-γ, but increases in Il-4 secretion ex vivo. The authors proposed that Hdac9 could act as an epigenetic switch in effector T cell-mediated systemic autoimmunity [69].

Periodontitis is a common and complex disease where the bacteria community engages an immune response that triggers tissue damage and bone loss [70]. Stem cells from periodontal ligament tissue (PDLSCs), when isolated from periodontitis patients, evidence a decreased osteogenic differentiation. Among all the zinc-dependent deacetylases evaluated, only HDAC9 is up-regulated in PDLSCs from these patients. Moreover, in PDLSCs *HDAC9* mRNA is up-regulated following TNF-α treatment. Silencing of HDAC9 seems to favor osteogenesis in vitro. Under this chronic inflammatory condition, a loop is sustained where *miR-17* and HDAC9 negatively affect each other [71]. It is possible that the different environmental conditions (inflammation) are responsible not only for the up-regulation of HDAC9, but also for eliciting specific influences on the deacetylase, in the context of the osteogenic differentiation. This consideration could explain the aforementioned pro-osteogenic role HDAC9/MITR in MSCs.

In mice, Hdac9 expression increases in the ischemic brain and its deletion protects from the ischemic reperfusion (I/R) injury. I/R triggers the up-regulation of several inflammatory genes which partially depends on Hdac9. A role of Hdac9 in the inflammatory response was confirmed by lipopolysaccharide treatment in cultured cells. Since the full activation of different signaling pathways, namely Erks, p38, Jnk, Nf-kb and Ikbα is dampened in the absence of Hdac9, it is plausible an action of the deacetylase as a very apical node [72]. Although the contribution of Hdac9 to the ischemic injury in the brain is evident, the molecular mechanism through which it exerts this action needs to be fully investigated.

In a different context an anti-inflammatory activity of HDAC9 was observed. Acne vulgaris is an inflammatory skin disorder. Bacterial products increase cytokine expression and histone acetylation in human sebocytes. Silencing of HDAC9 in these cells increases the expression of inflammatory cytokines (IL1B, CXCL8) particularly in the presence of MALP-2, a ligand for Toll-like receptors TLR2/6. Gene expression analysis confirmed that depletion of HDAC9 and also of HDAC8, increases the global inflammatory response in human sebocytes [73,74]. In contrast to other reports, this different cellular context points to HDAC9 as an epigenetic antagonist of the inflammatory response. The authors argued that the contribution of HDACs in the regulation of inflammation response could differ from inflammatory cells to other cell types. Indeed, deletion of HDAC9 and of other class IIa HDACs in non-inflammatory cells, similarly reported the up-regulation of some inflammatory cytokines [26,75]. The context-dependent effects of HDAC9 could be explained by the observation that class IIa are not required to completely abolish H3K27ac. Rather, they act as buffers on H3K27ac dynamic regions which are probably pre-determined in a lineage-specific manner [26,75].

HDAC9 is also involved in the regulation of the immune response. Regulatory T (T_reg_) lymphocytes are key factors for the maintenance of the peripheral self-tolerance. T_reg_ cells development and function is under the supervision of the TF FOXP3 [76]. Hdac9 is required for the regulation of the Foxp3-dependent immunosuppression. Levels of *Hdac9* mRNA were higher in Treg than in non-Treg cells and are up-regulated only in T_reg_ cells following T cell antigen receptor TCR stimulation. The proportions of CD4+ Foxp3+ T cells in lymphoid tissues of *Hdac9^-/-^* mice is augmented by ∼50% compared to wild-type mice. These cells are more active and show increased expression and acetylation of Foxp3 [77]. The role of Hdac9 as modulator of Treg functions was confirmed in a model of colitis. Hdac9 expression is increased in colon after induction of colitis and *Hdac9^-/-^* mice are resistant to colitis development [78].

TBK1 kinase plays a key role in the activation of the TF IRF3 and in the induction of type I interferons antiviral response [79]. In peritoneal macrophages knocked-out for the DNA methyltransferase *Dnmt3a*, the response to type I interferons is impaired. The abrogation of Dmnt3a activity results in the downregulation of Hdac9 expression. In this context Hdac9 influences the acetylation status of Tbk1, thus enhancing its kinase activity. Tbk1 activity is boosted by the wild-type Hdac9 but not by the Hdac9-∆C mutant [5].

In several studies that have unveiled a contribution of HDAC9 in a specific cellular response, as in the last example of the anti-viral signaling, the molecular mechanisms though which HDAC9 can exert its activity remain unknown. Whether HDAC9 is required to coordinate the assembly of specific multiprotein complexes and whether class I HDACs must be in these complexes require further investigation.

Finally, there is a growing excitement in combining epigenetic drugs with immunotherapy. The goal is to mount a more potent anti-cancer response. The complex role of HDAC9 in modulating different immune responses and particularly its influence on T_reg_ cell function, warrants further critical evaluations. If targeting HDAC9 and more in general class IIa HDACs in the context of an immunotherapeutic approach is planned, it will be important to exclude a possible negative effect.

## 6. Regulation of HDAC9 Expression and Cancer

*HDAC9* transcripts were frequently found up-regulated in cancer cells. For example, its expression can be sustained by oncogenes such as RAS [80]. By contrast, few information are available on the transcriptional networks controlling HDAC9 expression. During muscle differentiation MEF2D can sustain HDAC9 transcription in a negative feed-back circuit [81]. This feed-back mechanism is frequently circuited in tumors, [26,82,83]. Therefore, the MEF2 family members (MEF2A/B/C and D) are not only the foremost characterized partners of class IIa HDACs, but they can also fuel the transcription of these deacetylases. This circuit is responsible for the repression of the tumor suppressor gene Brahma/BRM, a SWI/SNF subunit dysregulated in different cancers and particularly in Rhabdoid sarcomas [83,84,85]. Likewise in leiomyosarcoma cells, a rare soft tissue tumor, MEF2D specifically upregulates the expression of HDAC9 by binding to its promoter region. Mutations of this binding motif strongly affect HDAC9 expression [26]. Importantly, in the same cells different MEF2 complexes, with opposite transcriptional outputs (activators and repressors) can co-exist, with the goal of fostering the transformed phenotype [82].

In muscle, oxygen levels play a role in modulating Hdac9 expression. Hypoxia up-regulates *Hdac9* mRNA and protein levels in C2C12 cells. This up-regulation sustains the hypoxia-dependent inhibition of muscle differentiation. Hdac9 can repress the transcription of some autophagy genes by the direct binding of the relative promoters. The authors propose that through this mechanism Hdac9 regulates autophagy and muscle differentiation in hypoxic conditions [86]. In the context of cancer progression, this environmental control on HDAC9 levels could provide an important contribution to the adaptation of the cancer cells to a hostile hypoxic environment.

Frequently, cancer cells are HDAC9-addicted, thus boosting further efforts in the identification of specific HDAC9 inhibitors [87,88]. Hdac9, is overexpressed in pancreatic ductal adenocarcinomas arising in mice expressing mutated Kras and lacking Rb. Hdac9 is highly expressed in tumor endothelial cells following the engagement of the Stat3 signaling pathway by the neoplastic cells [89]. High *HDAC9* mRNA levels are significantly associated with poor overall survival in medulloblastoma and glioblastoma (GBM) patients [90,91]. Silencing of HDAC9 reduces the proliferation of GBM cells and xenografts growth in vivo. EGFR (epidermal growth factor receptor) signaling is attenuated upon the down-regulation of HDAC9, through a mechanism that involves TAZ (Transcriptional coactivator with PDZ-binding motif) [91]. HDAC9 is highly expressed in human B-cell lymphomas and transgenic mice over-expressing Hdac9 develop, with age, lymphoproliferations and B-cell lymphomas [92]. High expression levels of HDAC9 and of other class IIa HDACs are significantly correlated with patient survival in hepatocellular carcinoma (HCC) [93]. In HCC cells HDAC9 levels are modestly up-regulated in response to TGF-β. When HDAC9 expression is down-regulated, sphere formation in culture is compromised. An indication of a role of HDAC9 in promoting anchorage-independent growth, as observed for other class IIa HDACs [76,94,95]. HCC cells abruptly up-regulate *HDAC9* mRNA in response to sulfatide a pro-metastatic sulfated glycolipid [96], further sustaining a role of HDAC9 in cancer onset and progression.

The relationships between HDAC9 levels and cancer are various and it can impact different steps of the tumorigenic process. MITR was identified among genes which expression is increased in chemo-resistant triple negative breast cancer cells. MITR confers paclitaxel resistance by releasing the repressive influence of MEF2A on IL11. However, the molecular mechanism responsible for such activation is obscure [97]. Another investigation revealed that in breast cancer full-length HDAC9 is responsible for the resistance to anti-estrogen treatments. High levels of HDAC9 are associated with worse prognosis in patients treated with the anti-estrogen tamoxifen. HDAC9 inhibits ERα expression and activity. Furthermore, among the different zinc-dependent HDACs, HDAC9 expression is dramatically increased in tamoxifen-resistant MCF7 cells and in ERα-negative breast tumor cell lines. In this context HDAC9 regulates the expression of gene sets related to tamoxifen resistance [98]. There are also cancers where HDAC9 expression is dysregulated at post-transcriptional levels. In esophageal squamous cell carcinoma the *miR-30d-5p* can modulate HDAC9 expression and the long non-coding (lncRNA) *LOC440173* acts as a sponge to sustain HDAC9 levels and the tumorigenic process [99].

In cutaneous squamous cell carcinomas, different SNPs in the *HDAC9* locus show a preferential allelic imbalance, thus suggesting that these variants could be selected during the tumorigenic process [100]. The *TWIST* locus is contiguous to *HDAC9* (Figure 1A) and it is unclear whether these SNPs affect HDAC9 activities or if they may influence TWIST1 expression, as distal regulative elements [101]. In fact, data analysis identified 12 enhancer candidates, located both within and outside the coding sequences of the *HDAC9*. Eight of these candidates are developmentally active during limb/fin and branchial arch formation in zebrafish, coincidentally with Twist expression. In an elegant study using 4C-seq, it was demonstrated that the *Twist1* promoter region interacts with three enhancers (eTw-5, 6, 7) in the limb bud and branchial arch of mouse embryos at day 11.5. CRISPR technology was used to prove the impact of these distal regulative elements on *Twist1* expression [102]. Alongside the role played by HDAC9 in regulating fitness [93], survival [26,103] and the metabolic reprogramming [104] of cancer cells, recent evidence reports a role of the deacetylase in regulating the tumor microenvironment and anti-tumor immunity. *Hdac9^-/-^* mice show decreased antitumor immunity in syngeneic models, as a consequence of decreased CD8^+^ dendritic cell tumor infiltration and, probably, the increased Foxp3+ infiltration [105]. Similar results were observed in clear-cell renal cell carcinoma, where HDAC9 promotes immune cell infiltration [106]. These results are in agreement with a previous report about the increased immunosuppressive properties of *Hdac9^-/-^* Tregs [78]. Most of these properties are due to the Hdac9-dependent inhibition of Mef2d, that in Tregs allows the acquisition of an effector Treg phenotype [107,108].

In summary, all the reported data on HDAC9 in cancer cells, although obtained in different models and in the presence of different stimuli, point to a pro-oncogenic activity for this deacetylase. However, it is important to note that the same epigenetic regulator can display opposite contributions during the transformation process, depending on the context. Although not clearly defined until now, a tumor suppressor function for HDAC9 cannot be excluded.

## 7. HDAC9 and DNA Damage

Epigenetic modifications occur following DNA damage and escort the DNA repair activities [107,108]. HDAC9 has been also implicated in DNA repair, a poorly investigated HDAC9-function that could be relevant in cancer progression. Ectopically expressed HDAC9 interacts with ATDC (ataxia telangiectasia group D-complementing) also known as TRIM29. ATDC is a member of the tripartite motif (TRIM) protein family, involved in the DNA damage response [109], that can impair TP53 activities. Instead, HDAC9 through the control of the acetylation status of ATDC, can inhibit ATDC-TP53 interaction, thus increasing the transcription activation function of TP53 [110]. A second study used siRNA-mediated down-regulation of different HDACs to unveil a specific contribution of HDAC9 to the homologous recombination process [111]. This preliminary observation does not clarify whether a direct or indirect (through the control of the expression of a specific target gene) engagement of HDAC9 is needed for an efficient DNA repair. Studies on HDAC9 and DNA repair are sporadic, but the importance of the biological implications deserves further attention.

## 8. Conclusions

**Figure 3:**
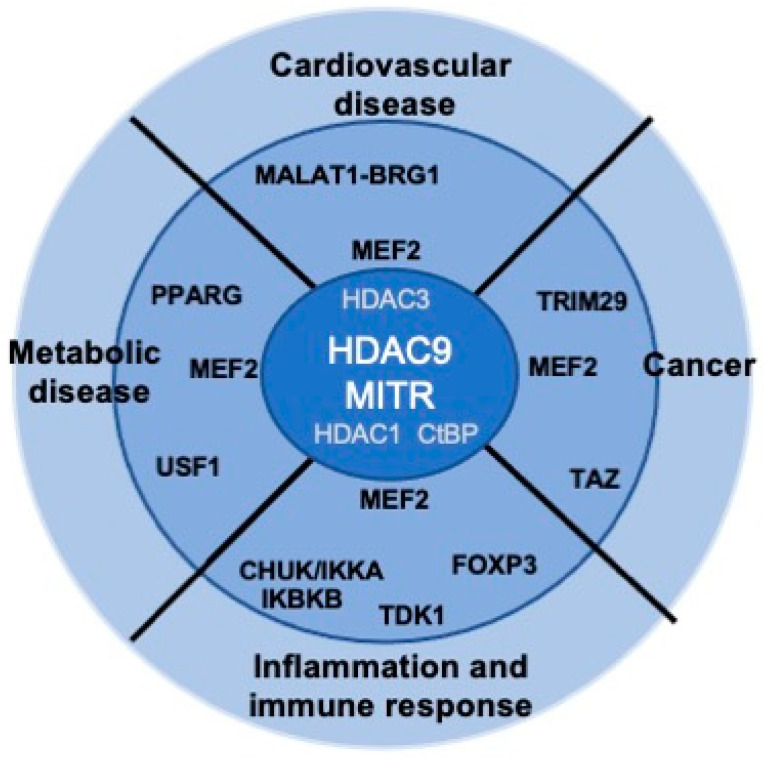
HDAC9 dysfunctions and human diseases. Some of the putative and described HDAC9 partners possibly involved in the different diseases are indicated.

Although HDAC9 was discovered more than two decades ago, several aspects of its activities remain poorly understood. The mechanisms governing HDAC9 expression have never been systematically addressed, including the regulation of its transcription and splicing (who control the controllers?). Even classical aspects of the regulation of HDAC9 activities, such as the control of the nuclear cytoplasmic shuttling, have been investigated only marginally. Given the important role of HDAC9 particularly in the cardiovascular system and in cancer (Figure 3), further studies aiming to characterize the complexity of this epigenetic regulator are strongly encouraged. We hope that the next decade will provide us with new information and, more importantly, with new compounds in order to modulate HDAC9 activities in a therapeutic perspective.

## References

[1] Lahm A., Paolini C., Pallaoro M., Nardi M.C., Jones P., Neddermann P., Sambucini S., Bottomley M.J., Lo Surdo P., Carfí A. (2007). Unraveling the hidden catalytic activity of vertebrate class IIa histone deacetylases. Proc. Natl. Acad. Sci. USA.

[2] Di Giorgio E., Brancolini C. (2016). Regulation of class IIa HDAC activities: It is not only matter of subcellular localization. Epigenomics.

[3] Clocchiatti A., Di Giorgio E., Demarchi F., Brancolini C. (2013). Beside the MEF2 axis: Unconventional functions of HDAC4. Cell Signal..

[4] Asfaha Y., Schrenk C., Alves Avelar L.A., Hamacher A., Pflieger M., Kassack M.U., Kurz T. (2019). Recent advances in class IIa histone deacetylases research. Bioorg. Med. Chem..

[5] Li X., Zhang Q., Ding Y., Liu Y., Zhao D., Zhao K., Shen Q., Liu X., Zhu X., Li N. (2016). Methyltransferase Dnmt3a upregulates HDAC9 to deacetylate the kinase TBK1 for activation of antiviral innate immunity. Nat. Immunol..

[6] Paroni G., Fontanini A., Cernotta N., Foti C., Gupta M.P., Yang X.J., Fasino D., Brancolini C. (2007). Dephosphorylation and caspase processing generate distinct nuclear pools of histone deacetylase 4. Mol. Cell Biol..

[7] Cernotta N., Clocchiatti A., Florean C., Brancolini C. (2011). Ubiquitin-dependent degradation of HDAC4, a new regulator of random cell motility. Mol. Biol. Cell..

[8] Lehmann L.H., Jebessa Z.H., Kreusser M.M., Horsch A., He T., Kronlage M., Dewenter M., Sramek V., Oehl U., Krebs-Haupenthal J. (2018). A proteolytic fragment of histone deacetylase 4 protects the heart from failure by regulating the hexosamine biosynthetic pathway. Nat. Med..

[9] Parra M. (2015). Class IIa HDACs-New insights into their functions in physiology and pathology. FEBS J..

[10] Parra M., Verdin E. (2010). Regulatory signal transduction pathways for class IIa histone deacetylases. Curr. Opin. Pharmacol..

[11] Paroni G., Cernotta N., Dello Russo C., Gallinari P., Pallaoro M., Foti C., Talamo F., Orsatti L., Steinkühler C., Brancolini C. (2008). PP2A regulates HDAC4 nuclear import. Mol. Biol. Cell.

[12] Sparrow D.B., Miska E.A., Langley E., Reynaud-Deonauth S., Kotecha S., Towers N., Spohr G., Kouzarides T., Mohun T.J. (1999). MEF-2 function is modified by a novel co-repressor, MITR. EMBO J..

[13] Zhou X., Richon V.M., Rifkind R.A., Marks P.A. (2000). Identification of a transcriptional repressor related to the noncatalytic domain of histone deacetylases 4 and 5. Proc. Natl. Acad. Sci. USA.

[14] Zhang C.L., McKinsey T.A., Lu J.R., Olson E.N. (2001). Association of COOH-terminal-binding protein (CtBP) and MEF2-interacting transcription repressor (MITR) contributes to transcriptional repression of the MEF2 transcription factor. J. Biol. Chem..

[15] Zhang C.L., McKinsey T.A., Olson E.N. (2001). The transcriptional corepressor MITR is a signal-responsive inhibitor of myogenesis. Proc. Natl. Acad. Sci. USA.

[16] Di Giorgio E., Clocchiatti A., Piccinin S., Sgorbissa A., Viviani G., Peruzzo P., Romeo S., Rossi S., Dei Tos A.P., Maestro R. (2013). MEF2 is a converging hub for histone deacetylase 4 and phosphatidylinositol 3-kinase/Akt-induced transformation. Mol. Cell Biol..

[17] Backs J., Backs T., Bezprozvannaya S., McKinsey T.A., Olson E.N. (2008). Histone deacetylase 5 acquires calcium/calmodulin-dependent kinase II responsiveness by oligomerization with histone deacetylase 4. Mol. Cell Biol..

[18] Zhou X., Marks P.A., Rifkind R.A., Richon V.M. (2001). Cloning and characterization of a histone deacetylase, HDAC9. Proc. Natl. Acad. Sci. USA.

[19] Mahlknecht U., Schnittger S., Will J., Cicek N., Hoelzer D. (2002). Chromosomal organization and localization of the human histone deacetylase 9 gene (HDAC9). Biochem. Biophys. Res. Commun..

[20] Petrie K., Guidez F., Howell L., Healy L., Waxman S., Greaves M., Zelent A. (2003). The histone deacetylase 9 gene encodes multiple protein isoforms. J. Biol. Chem..

[21] Zhang C.L., McKinsey T.A., Olson E.N. (2002). Association of class II histone deacetylases with heterochromatin protein 1: Potential role for histone methylation in control of muscle differentiation. Mol. Cell Biol..

[22] Zhang C.L., McKinsey T.A., Chang S., Antos C.L., Hill J.A., Olson E.N. (2002). Class II histone deacetylases act as signal-responsive repressors of cardiac hypertrophy. Cell.

[23] He T., Huang J., Chen L., Han G., Stanmore D., Krebs-Haupenthal J., Avkiran M., Hagenmüller M., Backs J. (2020). Cyclic AMP represses pathological MEF2 activation by myocyte-specific hypo-phosphorylation of HDAC5. J. Mol. Cell Cardiol..

[24] Harrison B.C., Huynh K., Lundgaard G.L., Helmke S.M., Perryman M.B., McKinsey T.A. (2010). Protein kinase C-related kinase targets nuclear localization signals in a subset of class IIa histone deacetylases. FEBS Lett..

[25] Hu S., Cho E.H., Lee J.Y. (2020). Histone Deacetylase 9: Its Role in the Pathogenesis of Diabetes and Other Chronic Diseases. Diabetes Metab. J..

[26] Di Giorgio E., Dalla E., Franforte E., Paluvai H., Minisini M., Trevisanut M., Picco R., Brancolini C. (2020). Different class IIa HDACs repressive complexes regulate specific epigenetic responses related to cell survival in leiomyosarcoma cells. Nucleic Acids Res..

[27] Clocchiatti A., Di Giorgio E., Viviani G., Streuli C., Sgorbissa A., Picco R., Cutano V., Brancolini C. (2015). The MEF2-HDAC axis controls proliferation of mammary epithelial cells and acini formation in vitro. J. Cell Sci..

[28] Chen Y.H., Yeh F.L., Yeh S.P., Ma H.T., Hung S.C., Hung M.C., Li L.Y. (2011). Myocyte enhancer factor-2 interacting transcriptional repressor (MITR) is a switch that promotes osteogenesis and inhibits adipogenesis of mesenchymal stem cells by inactivating peroxisome proliferator-activated receptor gamma-2. J. Biol. Chem..

[29] Jin Z., Wei W., Huynh H., Wan Y. (2015). HDAC9 Inhibits Osteoclastogenesis via Mutual Suppression of PPARγ/RANKL Signaling. Mol. Endocrinol..

[30] Chatterjee T.K., Idelman G., Blanco V., Blomkalns A.L., Piegore M.G., Weintraub D.S., Kumar S., Rajsheker S., Manka D., Rudich S.M. (2011). Histone deacetylase 9 is a negative regulator of adipogenic differentiation. J. Biol. Chem..

[31] Wong R.H., Chang I., Hudak C.S., Hyun S., Kwan H.Y., Sul H.S. (2009). A role of DNA-PK for the metabolic gene regulation in response to insulin. Cell.

[32] Li C.J., Cheng P., Liang M.K., Chen Y.S., Lu Q., Wang J.Y., Xia Z.Y., Zhou H.D., Cao X., Xie H. (2015). MicroRNA-188 regulates age-related switch between osteoblast and adipocyte differentiation. J. Clin. Investig..

[33] Chatterjee T.K., Basford J.E., Knoll E., Tong W.S., Blanco V., Blomkalns A.L., Rudich S., Lentsch A.B., Hui D.Y., Weintraub N.L. (2014). HDAC9 knockout mice are protected from adipose tissue dysfunction and systemic metabolic disease during high-fat feeding. Diabetes.

[34] Khamis A., Boutry R., Canouil M., Mathew S., Lobbens S., Crouch H., Andrew T., Abderrahmani A., Tamanini F., Froguel P. (2020). Histone deacetylase 9 promoter hypomethylation associated with adipocyte dysfunction is a statin-related metabolic effect. Clin. Epigenet..

[35] Lenoir O., Flosseau K., Ma F.X., Blondeau B., Mai A., Bassel-Duby R., Ravassard P., Olson E.N., Haumaitre C., Scharfmann R. (2011). Specific control of pancreatic endocrine β- and δ-cell mass by class IIa histone deacetylases HDAC4, HDAC5, and HDAC9. Diabetes.

[36] Chen J., Wang N., Dong M., Guo M., Zhao Y., Zhuo Z., Zhang C., Chi X., Pan Y., Jiang J. (2015). The Metabolic Regulator Histone Deacetylase 9 Contributes to Glucose Homeostasis Abnormality Induced by Hepatitis C Virus Infection. Diabetes.

[37] Chen J., Zhang Z., Wang N., Guo M., Chi X., Pan Y., Jiang J., Niu J., Ksimu S., Li J.Z. (2017). Role of HDAC9-FoxO1 Axis in the Transcriptional Program Associated with Hepatic Gluconeogenesis. Sci. Rep..

[38] Spracklen C.N., Karaderi T., Yaghootkar H., Schurmann C., Fine R.S., Kutalik Z., Preuss M.H., Lu Y., Wittemans L.B.L., Adair L.S. (2019). Exome-Derived Adiponectin-Associated Variants Implicate Obesity and Lipid Biology. Am. J. Hum. Genet..

[39] Méjat A., Ramond F., Bassel-Duby R., Khochbin S., Olson E.N., Schaeffer L. (2005). Histone deacetylase 9 couples neuronal activity to muscle chromatin acetylation and gene expression. Nat. Neurosci..

[40] Macpherson P.C., Farshi P., Goldman D. (2015). Dach2-Hdac9 signaling regulates reinnervation of muscle endplates. Development.

[41] Litke C., Bading H., Mauceri D. (2018). Histone deacetylase 4 shapes neuronal morphology via a mechanism involving regulation of expression of vascular endothelial growth factor D. J. Biol. Chem..

[42] Valzania A., Catale C., Viscomi M.T., Puglisi-Allegra S., Carola V. (2017). Histone deacetylase 5 modulates the effects of social adversity in early life on cocaine-induced behavior. Physiol. Behav..

[43] Sando R., Gounko N., Pieraut S., Liao L., Yates J., Maximov A. (2012). HDAC4 governs a transcriptional program essential for synaptic plasticity and memory. Cell.

[44] Taniguchi M., Carreira M.B., Cooper Y.A., Bobadilla A.C., Heinsbroek J.A., Koike N., Larson E.B., Balmuth E.A., Hughes B.W., Penrod R.D. (2017). HDAC5 and Its Target Gene, Npas4, Function in the Nucleus Accumbens to Regulate Cocaine-Conditioned Behaviors. Neuron.

[45] Woldemichael B.T., Jawaid A., Kremer E.A., Gaur N., Krol J., Marchais A., Mansuy I.M. (2016). The microRNA cluster miR-183/96/182 contributes to long-term memory in a protein phosphatase 1-dependent manner. Nat. Commun..

[46] Rahman M.S., Woollard K. (2017). Atherosclerosis. Adv. Exp. Med. Biol..

[47] Bellenguez C., Bevan S., Gschwendtner A., Spencer C.C., Burgess A.I., Pirinen M., Jackson C.A., Traylor M., International Stroke Genetics Consortium (ISGC), Wellcome Trust Case Control Consortium 2 (WTCCC2) (2012). Genome-wide association study identifies a variant in HDAC9 associated with large vessel ischemic stroke. Nat. Genet..

[48] Traylor M., Farrall M., Holliday E.G., Sudlow C., Hopewell J.C., Cheng Y.C., Fornage M., Ikram M.A., Malik R., Bevan S. (2012). Genetic risk factors for ischaemic stroke and its subtypes (the METASTROKE collaboration): A meta-analysis of genome-wide association studies. Lancet Neurol..

[49] Markus H.S., Mäkelä K.M., Bevan S., Raitoharju E., Oksala N., Bis J.C., O’Donnell C., Hainsworth A., Lehtimäki T. (2013). Evidence HDAC9 genetic variant associated with ischemic stroke increases risk via promoting carotid atherosclerosis. Stroke.

[50] Han Y., Sun W., Wang L., Tao S., Tian L., Hao Y., Zhang W., Wu S., Li S., Lv H. (2013). HDAC9 gene is associated with stroke risk in a Chinese population. Exp. Biol. Med..

[51] Dichgans M., Malik R., König I.R., Rosand J., Clarke R., Gretarsdottir S., Thorleifsson G., Mitchell B.D., Assimes T.L., Levi C. (2014). Shared genetic susceptibility to ischemic stroke and coronary artery disease: A genome-wide analysis of common variants. Stroke.

[52] Azghandi S., Prell C., van der Laan S.W., Schneider M., Malik R., Berer K., Gerdes N., Pasterkamp G., Weber C., Haffner C. (2015). Deficiency of the stroke relevant HDAC9 gene attenuates atherosclerosis in accord with allele-specific effects at 7p21.1. Stroke.

[53] Wang X.B., Han Y.D., Sabina S., Cui N.H., Zhang S., Liu Z.J., Li C., Zheng F. (2016). HDAC9 Variant Rs2107595 Modifies Susceptibility to Coronary Artery Disease and the Severity of Coronary Atherosclerosis in a Chinese Han Population. PLoS ONE.

[54] Shroff N., Ander B.P., Zhan X., Stamova B., Liu D., Hull H., Hamade F.R., Dykstra-Aiello C., Ng K., Sharp F.R. (2019). HDAC9 Polymorphism Alters Blood Gene Expression in Patients with Large Vessel Atherosclerotic Stroke. Transl. Stroke Res..

[55] Shi W., Wei X., Wang Z., Han H., Fu Y., Liu J., Zhang Y., Guo J., Dong C., Zhou D. (2016). HDAC9 exacerbates endothelial injury in cerebral ischaemia/reperfusion injury. J. Cell Mol. Med..

[56] Kuang S., Wang Z., Su L., Han X., Dong Q. (2018). Neuroprotection of histone deacetylase inhibitor TMP269 in cerebral ischemia/reperfusion rat. Int. J. Clin. Exp. Med..

[57] Nelson C.P., Goel A., Butterworth A.S., Kanoni S., Webb T.R., Marouli E., Zeng L., Ntalla I., Lai F.Y., Hopewell J.C. (2017). Association analyses based on false discovery rate implicate new loci for coronary artery disease. Nat. Genet..

[58] Matsukura M., Ozaki K., Takahashi A., Onouchi Y., Morizono T., Komai H., Shigematsu H., Kudo T., Inoue Y., Kimura H. (2015). Genome-Wide Association Study of Peripheral Arterial Disease in a Japanese Population. PLoS ONE.

[59] Klarin D., Lynch J., Aragam K., Chaffin M., Assimes T.L., Huang J., Lee K.M., Shao Q., Huffman J.E., Natarajan P. (2019). Genome-wide association study of peripheral artery disease in the Million Veteran Program. Nat. Med..

[60] Malhotra R., Mauer A.C., Lino Cardenas C.L., Guo X., Yao J., Zhang X., Wunderer F., Smith A.V., Wong Q., Pechlivanis S. (2019). HDAC9 is implicated in atherosclerotic aortic calcification and affects vascular smooth muscle cell phenotype. Nat. Genet..

[61] Prestel M., Prell-Schicker C., Webb T., Malik R., Lindner B., Ziesch N., Rex-Haffner M., Röh S., Viturawong T., Lehm M. (2019). The Atherosclerosis Risk Variant rs2107595 Mediates Allele-Specific Transcriptional Regulation of HDAC9 via E2F3 and Rb1. Stroke.

[62] Cao Q., Rong S., Repa J.J., St Clair R., Parks J.S., Mishra N. (2014). Histone deacetylase 9 represses cholesterol efflux and alternatively activated macrophages in atherosclerosis development. Arterioscler. Thromb. Vasc. Biol..

[63] Palm F., Aigner A., Pussinen P.J., Urbanek C., Buggle F., Safer A., Becher H., Grau A.J. (2020). Association of a Multigenetic Pro-Inflammatory Profile with Ischaemic Stroke. Cerebrovasc Dis..

[64] Chiou H.Y., Bai C.H., Lien L.M., Hu C.J., Jeng J.S., Tang S.C., Lin H.J., Hsieh Y.C. (2020). Interactive Effects of a Combination of the HDAC3 and HDAC9 Genes with Diabetes Mellitus on the Risk of Ischemic Stroke. Thromb. Haemost..

[65] Asare Y., Campbell-James T.A., Bokov Y., Yu L.L., Prestel M., El Bounkari O., Roth S., Megens R.T.A., Straub T., Thomas K. (2020). Histone Deacetylase 9 Activates IKK to Regulate Atherosclerotic Plaque Vulnerability. Circ. Res..

[66] Isselbacher E.M., Lino Cardenas C.L., Lindsay M.E. (2016). Hereditary Influence in Thoracic Aortic Aneurysm and Dissection. Circulation.

[67] Lino Cardenas C.L., Kessinger C.W., Cheng Y., MacDonald C., MacGillivray T., Ghoshhajra B., Huleihel L., Nuri S., Yeri A.S., Jaffer F.A. (2018). An HDAC9-MALAT1-BRG1 complex mediates smooth muscle dysfunction in thoracic aortic aneurysm. Nat. Commun..

[68] Lino Cardenas C.L., Kessinger C.W., Chou E.L., Ghoshhajra B., Yeri A.S., Das S., Weintraub N.L., Malhotra R., Jaffer F.A., Lindsay M.E. (2019). HDAC9 complex inhibition improves smooth muscle-dependent stenotic vascular disease. JCI Insight.

[69] Yan K., Cao Q., Reilly C.M., Young N.L., Garcia B.A., Mishra N. (2011). Histone deacetylase 9 deficiency protects against effector T cell-mediated systemic autoimmunity. J. Biol. Chem..

[70] Khan S.A., Kong E.F., Meiller T.F., Jabra-Rizk M.A. (2015). Periodontal Diseases: Bug Induced, Host Promoted. PLoS Pathog..

[71] Li L., Liu W., Wang H., Yang Q., Zhang L., Jin F., Jin Y. (2018). Mutual inhibition between HDAC9 and miR-17 regulates osteogenesis of human periodontal ligament stem cells in inflammatory conditions. Cell Death Dis..

[72] Lu S., Li H., Li K., Fan X.D. (2018). HDAC9 promotes brain ischemic injury by provoking IκBα/NF-κB and MAPKs signaling pathways. Biochem. Biophys. Res. Commun..

[73] Sanford J.A., Zhang L.J., Williams M.R., Gangoiti J.A., Huang C.M., Gallo R.L. (2016). Inhibition of HDAC8 and HDAC9 by microbial short-chain fatty acids breaks immune tolerance of the epidermis to TLR ligands. Sci. Immunol..

[74] Sanford J.A., O’Neill A.M., Zouboulis C.C., Gallo R.L. (2019). Short-Chain Fatty Acids from Cutibacterium acnes Activate Both a Canonical and Epigenetic Inflammatory Response in Human Sebocytes. J. Immunol..

[75] Cutano V., Di Giorgio E., Minisini M., Picco R., Dalla E., Brancolini C. (2019). HDAC7-mediated control of tumour microenvironment maintains proliferative and stemness competence of human mammary epithelial cells. Mol. Oncol..

[76] Terry L.V., Oo Y.H. (2020). The Next Frontier of Regulatory T Cells: Promising Immunotherapy for Autoimmune Diseases and Organ Transplantations. Front. Immunol..

[77] Tao R., de Zoeten E.F., Ozkaynak E., Chen C., Wang L., Porrett P.M., Li B., Turka L.A., Olson E.N., Greene M.I. (2007). Deacetylase inhibition promotes the generation and function of regulatory T cells. Nat. Med..

[78] de Zoeten E.F., Wang L., Sai H., Dillmann W.H., Hancock W.W. (2010). Inhibition of HDAC9 increases T regulatory cell function and prevents colitis in mice. Gastroenterology.

[79] Cao X. (2016). Self-regulation and cross-regulation of pattern-recognition receptor signalling in health and disease. Nat. Rev. Immunol..

[80] Mason D.X., Jackson T.J., Lin A.W. (2004). Molecular signature of oncogenic RAS-induced senescence. Oncogene.

[81] Haberland M., Arnold M.A., McAnally J., Phan D., Kim Y., Olson E.N. (2007). Regulation of HDAC9 gene expression by MEF2 establishes a negative-feedback loop in the transcriptional circuitry of muscle differentiation. Mol. Cell Biol..

[82] Di Giorgio E., Franforte E., Cefalù S., Rossi S., Dei Tos A.P., Brenca M., Polano M., Maestro R., Paluvai H., Picco R. (2017). The co-existence of transcriptional activator and transcriptional repressor MEF2 complexes influences tumor aggressiveness. PLoS Genet..

[83] Di Giorgio E., Hancock W.W., Brancolini C. (2018). MEF2 and the tumorigenic process, hic sunt leones. Biochim. Biophys. Acta Rev. Cancer.

[84] Kahali B., Gramling S.J., Marquez S.B., Thompson K., Lu L., Reisman D. (2014). Identifying targets for the restoration and reactivation of BRM. Oncogene.

[85] Kahali B., Yu J., Marquez S.B., Thompson K.W., Liang S.Y., Lu L., Reisman D. (2014). The silencing of the SWI/SNF subunit and anticancer gene BRM in Rhabdoid tumors. Oncotarget.

[86] Zhang Z., Zhang L., Zhou Y., Li L., Zhao J., Qin W., Jin Z., Liu W. (2019). Increase in HDAC9 suppresses myoblast differentiation via epigenetic regulation of autophagy in hypoxia. Cell Death Dis..

[87] Di Giorgio E., Gagliostro E., Brancolini C. (2015). Selective class IIa HDAC inhibitors: Myth or reality. Cell Mol. Life Sci..

[88] Porter N.J., Christianson D.W. (2019). Structure, mechanism, and inhibition of the zinc-dependent histone deacetylases. Curr. Opin. Struct. Biol..

[89] Gore J., Craven K.E., Wilson J.L., Cote G.A., Cheng M., Nguyen H.V., Cramer H.M., Sherman S., Korc M. (2015). TCGA data and patient-derived orthotopic xenografts highlight pancreatic cancer-associated angiogenesis. Oncotarget.

[90] Milde T., Oehme I., Korshunov A., Kopp-Schneider A., Remke M., Northcott P., Deubzer H.E., Lodrini M., Taylor M.D., von Deimling A. (2010). HDAC5 and HDAC9 in medulloblastoma: Novel markers for risk stratification and role in tumor cell growth. Clin. Cancer Res..

[91] Yang R., Wu Y., Wang M., Sun Z., Zou J., Zhang Y., Cui H. (2015). HDAC9 promotes glioblastoma growth via TAZ-mediated EGFR pathway activation. Oncotarget.

[92] Gil V.S., Bhagat G., Howell L., Zhang J., Kim C.H., Stengel S., Vega F., Zelent A., Petrie K. (2016). Deregulated expression of HDAC9 in B cells promotes development of lymphoproliferative disease and lymphoma in mice. Dis. Models Mech..

[93] Freese K., Seitz T., Dietrich P., Lee S.M.L., Thasler W.E., Bosserhoff A., Hellerbrand C. (2019). Histone Deacetylase Expressions in Hepatocellular Carcinoma and Functional Effects of Histone Deacetylase Inhibitors on Liver Cancer Cells In Vitro. Cancers.

[94] Kanki K., Watanabe R., Nguyen Thai L., Zhao C.H., Naito K. (2020). HDAC9 Is Preferentially Expressed in Dedifferentiated Hepatocellular Carcinoma Cells and Is Involved in an Anchorage-Independent Growth. Cancers.

[95] Paluvai H., Di Giorgio E., Brancolini C. (2018). Unscheduled HDAC4 repressive activity in human fibroblasts triggers TP53-dependent senescence and favors cell transformation. Mol. Oncol..

[96] Dong Y.W., Wang R., Cai Q.Q., Qi B., Wu W., Zhang Y.H., Wu X.Z. (2014). Sulfatide epigenetically regulates miR-223 and promotes the migration of human hepatocellular carcinoma cells. J. Hepatol..

[97] Lian B., Pei Y.C., Jiang Y.Z., Xue M.Z., Li D.Q., Li X.G., Zheng Y.Z., Liu X.Y., Qiao F., Sun W.L. (2020). Truncated HDAC9 identified by integrated genome-wide screen as the key modulator for paclitaxel resistance in triple-negative breast cancer. Theranostics.

[98] Linares A., Assou S., Lapierre M., Thouennon E., Duraffourd C., Fromaget C., Boulahtouf A., Tian G., Ji J., Sahin O. (2019). Increased expression of the HDAC9 gene is associated with antiestrogen resistance of breast cancers. Mol. Oncol..

[99] Wang G., Feng B., Niu Y., Wu J., Yang Y., Shen S., Guo Y., Liang J., Guo W., Dong Z. (2020). A novel long noncoding RNA, LOC440173, promotes the progression of esophageal squamous cell carcinoma by modulating the miR-30d-5p/HDAC9 axis and the epithelial-mesenchymal transition. Mol. Carcinog..

[100] Fleming J.L., Dworkin A.M., Allain D.C., Fernandez S., Wei L., Peters S.B., Iwenofu O.H., Ridd K., Bastian B.C., Toland A.E. (2014). Allele-specific imbalance mapping identifies HDAC9 as a candidate gene for cutaneous squamous cell carcinoma. Int. J. Cancer.

[101] Siekmann T.E., Gerber M.M., Toland A.E. (2016). Variants in an Hdac9 intronic enhancer plasmid impact Twist1 expression in vitro. Mamm. Genome.

[102] Hirsch N., Eshel R., Bar Yaacov R., Shahar T., Shmulevich F., Dahan I., Levaot N., Kaplan T., Lupiáñez D.G., Birnbaum R.Y. (2018). Unraveling the transcriptional regulation of TWIST1 in limb development. PLoS Genet..

[103] Kai X., Hejun Z., Yang D., Jie T., Shigang D. (2019). Identification of HDAC9 as a viable therapeutic target for the treatment of gastric cancer. Exp. Mol. Med..

[104] Peruzzo P., Comelli M., Di Giorgio E., Franforte E., Mavelli I., Brancolini C. (2016). Transformation by different oncogenes relies on specific metabolic adaptations. Cell Cycle.

[105] Di Giorgio E., Wang L., Xiong Y., Akimova T., Christensen L.M., Han R., Samanta A., Trevisanut M., Bhatti T.R., Beier U.H. (2020). MEF2D sustains activation of effector Foxp3+ Tregs during transplant survival and anticancer immunity. J. Clin. Investig..

[106] Ning Y., Ding J., Sun X., Xie Y., Su M., Ma C., Pan J., Chen J., Jiang H., Qi C. (2020). HDAC9 deficiency promotes tumor progression by decreasing the CD8 + dendritic cell infiltration of the tumor microenvironment. J. Immunother. Cancer.

[107] Van H.T., Santos M.A. (2018). Histone modifications and the DNA double-strand break response. Cell Cycle.

[108] Paluvai H., Di Giorgio E., Brancolini C. (2020). The Histone Code of Senescence. Cells.

[109] Yang H., Palmbos P.L., Wang L., Kim E.H., Ney G.M., Liu C., Prasad J., Misek D.E., Yu X., Ljungman M. (2015). ATDC (Ataxia Telangiectasia Group D Complementing) Promotes Radioresistance through an Interaction with the RNF8 Ubiquitin Ligase. J. Biol. Chem..

[110] Yuan Z., Peng L., Radhakrishnan R., Seto E. (2010). Histone deacetylase 9 (HDAC9) regulates the functions of the ATDC (TRIM29) protein. J. Biol. Chem..

[111] Kotian S., Liyanarachchi S., Zelent A., Parvin J.D. (2011). Histone deacetylases 9 and 10 are required for homologous recombination. J. Biol. Chem..

